# Towards Tamper-Proof Trust Evaluation of Internet of Things Nodes Leveraging IOTA Ledger

**DOI:** 10.3390/s25154697

**Published:** 2025-07-30

**Authors:** Assiya Akli, Khalid Chougdali

**Affiliations:** Engineering Sciences Laboratory, Ibn Tofail University, Kenitra 14000, Morocco; khalid.chougdali@uit.ac.ma

**Keywords:** IOTA tangle, IoT, trust evaluation

## Abstract

Trust evaluation has become a major challenge in the quickly developing Internet of Things (IoT) environment because of the vulnerabilities and security hazards associated with networked devices. To overcome these obstacles, this study offers a novel approach for evaluating trust that uses IOTA Tangle technology. By decentralizing the trust evaluation process, our approach reduces the risks related to centralized solutions, including privacy violations and single points of failure. To offer a thorough and reliable trust evaluation, this study combines direct and indirect trust measures. Moreover, we incorporate IOTA-based trust metrics to evaluate a node’s trust based on its activity in creating and validating IOTA transactions. The proposed framework ensures data integrity and secrecy by implementing immutable, secure storage for trust scores on IOTA. This ensures that no node transmits a wrong trust score for itself. The results show that the proposed scheme is efficient compared to recent literature, achieving up to +3.5% higher malicious node detection accuracy, up to 93% improvement in throughput, 40% reduction in energy consumption, and up to 24% lower end-to-end delay across various network sizes and adversarial conditions. Our contributions improve the scalability, security, and dependability of trust assessment processes in Internet of Things networks, providing a strong solution to the prevailing issues in current centralized trust models.

## 1. Introduction

The Internet of Things (IoT) represents a transformative evolution in digital technology, characterized by the interconnection of a vast array of devices and systems, ranging from everyday household items to sophisticated industrial machinery [1]. This widespread integration has fueled major advances in sectors such as smart healthcare, intelligent transportation, energy optimization, and industrial automation [2]. These applications rely heavily on the seamless exchange of data and autonomous decision-making. However, as the number of connected devices continues to grow, ensuring that all participating nodes are reliable and secure becomes a foundational concern [3]. The presence of malicious and compromised nodes in the network can lead to security breaches, data corruption, and system malfunctions, which poses severe risks to individuals and organizations [4]. Therefore, robust mechanisms for trust evaluation within IoT networks are essential to maintaining the integrity, security, and reliability of these interconnected systems [5].

Evaluating the trustworthiness of network nodes in IoT is paramount to safeguarding the network’s overall integrity and functionality [6]. Trust evaluation mechanisms help in identifying and isolating malicious or faulty nodes to prevent potential security risks including network failures, unauthorized access, and data breaches [7]. Recent literature has proposed various solutions to address these challenges. Traditional centralized trust management systems rely on a central authority to assess and validate trust, but these models are exposed to single points of failure and scalability issues [8]. Alternatively, distributed trust management approaches have gained traction, leveraging peer-to-peer interactions and reputation systems to evaluate trust. Some studies have explored the use of machine learning algorithms to dynamically assess node behavior and predict trustworthiness [9]. However, these methods often face challenges related to computational overhead and the requirement for real-time data processing [10]. More recent advancements include the adoption of blockchain and distributed ledger technologies to create immutable and transparent trust evaluation frameworks to enhance security and resilience against tampering and fraud [6,11]. Despite these innovations, there remains a need for more efficient, scalable, and secure trust evaluation mechanisms that can seamlessly integrate with the diverse and dynamic nature of IoT networks.

Trust refers to the confidence that one node places in another node within the network to behave predictably and reliably based on its past behavior and interactions [12]. We define trust as a dynamic, context-dependent concept that evolves over time, driven by the node’s historical performance and interactions. A node’s trustworthiness is determined by its reliability in providing services (such as data transmission, validation of transactions, etc.), its responsiveness to requests, and its adherence to expected behaviors [13,14].

The need for a decentralized trustworthiness evaluation mechanism in IoT networks has become increasingly evident as the limitations of centralized systems are more clearly understood. Traditional trust paradigms typically rely on centralized cluster heads to evaluate and disseminate trust information, which creates a single point of failure [15,16] that can be exploited by malicious actors to disrupt or manipulate network operations. Such centralized architectures also raise significant privacy concerns, as sensitive trust data must be aggregated and processed by a central authority, increasing the risk of unauthorized access or misuse [17]. Furthermore, these models often lack standardized and quantitative trust metrics, which leads to subjective assessments that hinder consistent and reliable decision-making across heterogeneous networks [18].

In contrast, decentralized trust evaluation approaches eliminate reliance on a single authority by distributing trust computation and decision-making across participating nodes. This distribution enhances system resilience, improves scalability, and better preserves privacy. Decentralized models are inherently more robust against targeted attacks, such as trust manipulation or data interception, as trust is derived from local observations and peer consensus. However, they introduce challenges related to synchronization, latency, and the need for efficient consensus protocols [16]. Despite these trade-offs, the comparative advantages of decentralization, particularly in terms of fault tolerance and data integrity, underscore the urgent need for a paradigm shift toward decentralized and tamper-resistant trust evaluation frameworks tailored to the dynamic nature of IoT environments.

A key requirement for any decentralized trust evaluation framework is the prevention of fraudulent trust scores [19]. In a decentralized system, it is crucial that no node manipulates its own trust score or the scores of other nodes to present a fake trust value. This ensures the reliability and integrity of the trust evaluation process. Leveraging distributed ledger technologies, such as IOTA, can address this challenge by providing a secure and immutable record of trust scores [20]. Each transaction is cryptographically secured and recorded on the ledger, making it tamper-proof. This immutability ensures that once a trust score is assigned, it cannot be altered or forged by any node to maintain the authenticity of the trust evaluation process. Such a robust mechanism is essential for building a secure and trustworthy IoT ecosystem, where each node’s behavior can be accurately assessed and monitored.

To address these pressing concerns, this paper proposes a novel approach to trust evaluation within IoT networks, aimed at mitigating the vulnerabilities inherent in centralized architectures. Our approach decentralizes the trust assessment process, distributing the responsibility of trust evaluation across multiple nodes. Central to our approach is the development of mechanisms to ensure the integrity and confidentiality of sensitive trust data stored on IoT nodes. We leverage IOTA Tangle to create an immutable and secure repository for trust scores to ensure that once trust data are recorded, they cannot be tampered with or altered by malicious actors. This cryptographic security fortifies the overall security posture of the system, as trust scores remain authentic and reliable. Additionally, by decentralizing the storage and management of trust data, the proposed scheme minimizes the risk of privacy breaches. Each node contributes to the trust evaluation process without needing to disclose sensitive information to a central authority, thereby preserving the privacy of individual nodes and preventing a single point of failure.

The proposed framework also incorporates both direct and indirect trust metrics, providing a comprehensive and nuanced assessment of each node’s trustworthiness. Direct metrics include the node’s own behavior and historical performance, while indirect metrics are derived from feedback and interactions with other nodes in the network. In addition, the framework introduces a third dimension: IOTA-based behavioral metrics, including Transaction Confirmation Rate (TCR), Reattachment Frequency (RF), Cumulative Weight Contribution (CWC), and Cumulative Transaction Contribution (CTC). By integrating these diverse metrics, we achieve a more accurate and holistic evaluation of trust, enhancing the network’s ability to detect and isolate malicious or unreliable nodes. This multi-faceted approach not only strengthens security but also ensures the system’s robustness and reliability. This paper contributes the following:

- Development of a decentralized trust evaluation framework utilizing IOTA technology to mitigate risks associated with centralized trust evaluation methods, such as single points of failure and privacy breaches.
- Incorporation of IOTA-based trust metrics, which evaluate a node’s activity in creating and validating transactions on the Tangle. This enhances the accuracy and resilience of trust computation.
- Implementation of secure and immutable storage for trust scores, ensuring data integrity and confidentiality.

The remainder of this paper is structured as follows: Section 2 examines recent literature on trust evaluation for IoT networks. In Section 4, we detail the proposed decentralized trust evaluation framework to highlight its design principles and implementation. The experimental setup and results, which are detailed in Section 5, showcase the framework’s effectiveness. Finally, this paper concludes and suggests potential directions for future research in Section 6.

## 2. Literature Review

In this section, we review the recent literature that evaluates node trust in IoT. The literature is divided intro three categories: direct and indirect trust assessments, centralized methods, and decentralized methods to evaluate trust. A summary of the literature reviewed is given in Table 1.

### 2.1. Direct and Indirect Metrics for Trust Assessment

Trust assessment in IoT systems leverages both direct and indirect metrics to enhance reliability and security.

Altaf et al. [21] proposed a Context-Based Trust Evaluation System (CTES) for the Industrial Internet of Things (IIoT), which calculates trust scores for edge nodes by combining direct trust from user satisfaction scores and indirect trust from node recommendations. The system dynamically adjusts the weights of these trust components based on contextual factors, aiming to filter out malicious nodes effectively. In [22], the authors introduce a framework called the Intelligent Trust Collaboration Network System (ITCN), which employs mobile vehicles and UAVs for decentralized data collection in IoT networks. The system uses a trust evaluation mechanism that compares data collected by UAVs and vehicles, optimizing data collection efficiency while maintaining security through direct and indirect trust metrics.

Djedjig et al. [23] present a trust evaluation method that integrates direct trust metrics, such as honesty and energy efficiency, with indirect trust derived from neighboring node recommendations. This approach continuously updates and propagates trust values to enhance the security and reliability of IoT network routing. The authors of [24] develop a T-Safe framework for evaluating sensor node trustworthiness in intelligent transport systems. The framework assesses trust through direct metrics like security and data quality, combined with indirect trust from referrals by neighboring nodes. Trust values are updated using a sliding window approach to ensure accurate and reliable service provisioning.

The model proposed by [30] evaluates entities using a combination of multi-context quality of service, quality of provider, and market value. Trust is calculated dynamically through the IoT-TESM algorithm, which uses an auto-scale weighting (ASW) technique to adjust trust metrics over time. The model outperforms other frameworks like ReGreT and SIoT in selecting reliable service providers. However, limitations include the need for extensive contextual data, potential complexity in deployment, and incomplete evaluation against adversarial attacks in highly adversarial or data-scarce settings.

While these approaches demonstrate the effectiveness of integrating direct and indirect trust metrics, it is crucial to know that expanding the range of metrics can impact system complexity and computational resources, particularly in dynamic and large-scale IoT environments. Additionally, the need for synchronization and continuous updates affect real-time performance and scalability.

### 2.2. Centralized and Semi-Centralized Trust Evaluation Methods

Centralized and semi-centralized trust evaluation methods offer structured and coordinated approaches to managing trust in IoT systems.

The authors of [31] propose a centralized trust evaluation mechanism for Underwater Wireless Sensor Networks (UWSNs) called TEUC. This system utilizes the C4.5 decision tree algorithm to classify nodes based on multi-dimensional trust evidence, enabling better detection of malicious nodes and efficient energy consumption. In [32], a lightweight centralized trust management system is proposed for IoT-based edge nodes called lightTrust. The system divides trust management into Trust Agent and Trust Developer components, where trust is evaluated based on parameters like compatibility, cooperativeness, and delivery ratio, ensuring secure communication among edge nodes.

Liu et al. [25] present a semi-centralized trust management model that integrates blockchain technology with centralized cloud servers. This model combines direct trust from historical interactions with indirect trust from recommendations, achieving a balance between the efficiency of centralization and the security of decentralization. A hybrid trust management model for IIoT environments has been proposed in [26], which combines centralized and distributed approaches. Trust is managed within clusters, with continuous updates to ensure real-time accuracy. This hybrid model addresses scalability and energy consumption while enhancing the reliability of trust metrics.

Although they provide structured and coordinated frameworks for managing trust in IoT environments, centralized and semi-centralized methods may encounter issues with scalability, adaptability, and complexity, as well as security and privacy.

### 2.3. Decentralized Trust Management Algorithms

Decentralized trust management algorithms address the challenges of scalability, flexibility, and robustness in IoT systems by eliminating the need for a central authority.

A trust evaluation model has been proposed in [33] for IoT networks to address challenges posed by centralized trust mechanisms. This scheme leverages mobile edge nodes to collect, calculate, and relay trust information between sensor nodes and cloud platforms using trust chains and an improved Dijkstra’s algorithm. The model introduces compositional trust chain evaluation (atomic, tandem, parallel) and incorporates multiple trust components like interaction trust, energy trust, and recommendation trust.

In [27], the authors combine blockchain with trust evaluation to address vulnerabilities in access control. The proposed system forms blockchain-based IIoT groups (B-IIoT-G), where IIoT devices maintain access control lists (ACLs) and vote on authorization decisions using trust-weighted voting mechanisms rather than majority rule alone. The system introduces a lightweight blockchain structure to reduce storage and processing demands and updates trust values dynamically based on historical behaviors and voting outcomes.

A framework called BC-Trust has been introduced in [34]. It is a blockchain-based trust evaluation method that leverages fog computing. This system offloads trust information storage and computation to fog nodes, ensuring scalable and secure trust assessments across IoT devices. A decentralized Attribute-Based Access Control (ABAC) system integrated with a Trust and Reputation System (TRS) has been proposed in [35]. Trust and reputation scores are calculated via smart contracts on a public blockchain, supporting dynamic access control and enhanced security in IoT networks.

Xiao et al. [36] developed a decentralized trust management system (DTMS) that combines blockchain technology with a consensus-based model. The system utilizes a hierarchical design to perform trust evaluations locally, ensuring transparency and tamper resistance while rewarding honest participation. The authors of [29] proposed the BD-VTE scheme, a decentralized trust evaluation method for smart network systems. The scheme uses Mobile Vehicles (MVs) and UAVs for active data verification, enhancing the reliability and efficiency of data collection through distributed trust assessment.

These decentralized approaches offer innovative solutions for managing trust in IoT systems, enhancing scalability, flexibility, and security. Nevertheless, these proposed algorithms may introduce latency, resource demands, and implementation complexity [37,38].

## 3. Network Model

**Figure 1:**
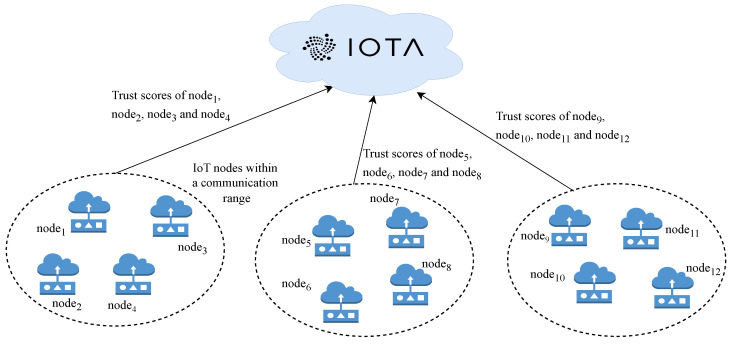
Entities and flow of data in the proposed system model.

The system model is shown in Figure 1. The entities in the system model include IoT sensor nodes and IOTA Tangle. The nodes within each other’s communication range evaluate trust for every other node. Once a trust value is assigned to each node and a consensus is achieved, these trust values are stored on the immutable IOTA network, which is accessible to all the nodes within the IoT network.

### Adversary Model

We consider an adversary that aims to manipulate trust scores, disrupt network operations, or degrade trust evaluation accuracy. The following attack scenarios are considered:

- Sybil Attack: Malicious entities create multiple fake identities (Sybil nodes) to overwhelm the network, manipulate trust scores, and gain an unfair advantage. This leads to trust dilution and misleading reputation values.
- Self-Promotion Attack: A malicious node falsely increases its own trust score by injecting fake transactions or repeatedly reattaching its own transactions to the Tangle. This can compromise decision-making in the trust evaluation process.
- Bad-Mouthing Attack: Malicious nodes deliberately assign low trust scores to legitimate nodes to reduce their reputation and cause network disruption. This can result in honest nodes being falsely labeled as untrustworthy.
- Collusion Attack: A group of compromised nodes coordinate to unfairly boost each other’s trust scores while degrading others. This introduces bias in trust evaluation and allows attackers to manipulate network behavior.
- Resource Depletion Attack (DoS on Trust Evaluation): Attackers attempt to overload the trust evaluation process by flooding the network with excessive transaction verification requests. This increases computation and communication costs.

## 4. Proposed Methodology

The proposed scheme consists of two phases: a decentralized and scalable collaborative trust evaluation framework and ledger integration for an immutable storage of sensitive records. In the first phase, the nodes start communicating with each other, initially broadcasting their location data. The messages contain a node’s ID, location, message-broadcast timestamp, and initial trust score, which is zero. The message is given as follows:

$$Message_{i}=\left\{ID_{i},location_{i},msg\_timestamp,TS_{i}\right\}$$

For each node *i*, these messages are received by the nodes within its communication range. Once other nodes have these data, they perform computations to evaluate the trustworthiness of node *i*.

The proposed framework fuses both direct and indirect trust metrics. Direct metrics include node reliability and QoS to capture a node’s individual performance and communication behavior. Indirect trust emerges from the node’s position within the network (centrality) and its contribution to IOTA’s consensus mechanisms. These are combined into a local trust score using an exponential weighting scheme (Equation (14)), which emphasizes high performance. Meanwhile, IOTA-based trust metrics form a decentralized trust score (ITS) based on immutable transaction activity (Equation (13)). The final trust score (Equation (15)) blends these scores using the tunable parameter λ.

### 4.1. Node Reliability

The reliability of a node is computed based on how reliable a node is in terms of communications with other nodes. Nodes that have high efficiency with regard to communications have a high reliability score. To compute the reliability of a node *i*, the message-receiving nodes determine communication consistency and bit error rate.

#### 4.1.1. Communication Consistency

Communication consistency measures the consistency of a node in terms of communication patterns. Nodes with inconsistent communication patterns are considered less reliable. It is computed using the following equation:

(1) $$CC_{i}=1-\frac{\sigma (T_{i})}{\mu (T_{i})}$$

where $CC_{i}$ is the communication consistency of node *i*, $\sigma (T_{i})$ is the standard deviation, and $\mu (T_{i})$ is the mean of the time intervals $T_{i}$ between successive communications of node *i*.

#### 4.1.2. Bit Error Rate (BER)

BER is a statistical measure that quantifies the percentage of bits that are received incorrectly in a digital communication system. It is computed as follows:

(2) $$BER_{i}=\frac{Bit\;errors}{Total\;bits}$$

where $BER_{i}$ is the bit error rate of node *i*. Node trust relies on the ability to detect errors in data transmission. By measuring BER, we assess the effectiveness of error detection in place.

#### 4.1.3. Reliability Score

Once communication consistency and bit error rate are computed, the message-receiving node computes reliability. Reliability computation depends on a weight called ρ. It controls the relative importance of CC versus BER. This parameter ranges from 0 to 1, where higher values emphasize CC, which is vital in environments where communication frequency is a strong indicator of node reliability. Occasional bit errors can be corrected, but erratic communication patterns signal unreliable and potentially malicious behavior. Therefore, ρ is set empirically to reflect that CC is more indicative of long-term trustworthiness. Reliability is computed as follows:

(3) $$reliability_{i}=\rho \times CC_{i}+\left(\rho -1\right)\times BER_{i}$$

The weight ρ determines the importance of the metrics $CC_{i}$ and $BER_{i}$ and 0≤ρ≤1.

### 4.2. Quality of Service Measures

Quality of Service (QoS) metrics determine the efficiency and effectiveness of a node. These metrics indicate a node’s performance, which directly impacts its trustworthiness. The QoS evaluation comprises three primary parameters: latency, throughput, and jitter. Each of these parameters offers a distinct perspective on the service quality of a node. Nodes that consistently offer high QoS are considered more trustworthy. Before computing QoS metrics, each node measures latency and throughput for node *i*.

#### 4.2.1. Latency

Latency is a measure of the time it takes for a message to travel from its source to its destination. We consider latency to measure QoS in order to ensure timely delivery of important messages. Lower latency values are indicative of faster communication and more efficient network operations.

The average latency $L_{i}$ from node *i* to all other nodes is expressed as follows:

(4) $$L_{i}=\frac{1}{N-1}\sum_{\begin{array}{c} j\neq i \end{array}}T_{i,j}$$

where $T_{i,j}$ is the time taken for a message to travel from node *i* to node *j*, and *N* is the total number of nodes in the network.

In Equation (4), the summation $\sum_{\begin{array}{c} j\neq i \end{array}}T_{i,j}$ calculates the total travel time for messages from node *i* to all other nodes *j*, and N−1 measures the number of other nodes (excluding node *i* itself).

#### 4.2.2. Throughput

Throughput refers to the total data transmitted by a node over the total time. It measures the data handling capacity of the node, which provides insights into its capability to process and transmit large volumes of data efficiently. Higher throughput values denote a node’s robust data management and transfer abilities. It is computed using the following equation:

(5) $$Throughput_{i}=\frac{Total\;data\;transmitted}{Total\;time}$$

In the above equation, throughput is the rate at which data are transmitted, total data transmitted is the amount of data sent during the measurement period, and total time is the duration of the measurement period.

#### 4.2.3. Jitter

Jitter is the variation in transmission delays among two packets in a packet stream, without taking into consideration the possible lost packets. Transmission delays can be altered by network characteristics such as poor connections, node breakdowns, and collisions. A decrease in the latency time suggests the presence of an attack on the network. We compute jitter using the following equation:

(6) $$Jitter=\frac{1}{N-1}\sum_{i=1}^{P-1}\left|\left(D_{i+1}-D_{i}\right)\right|$$

where *P* is the total number of packets and $D_{i}$ is the transmission delay of the $i^{\mathrm{th}}$ packet.

Once latency, throughput, and jutter have been computed for node *i*, a final QoS score is computed.

#### 4.2.4. QoS Score

The final QoS score is computed using a weighted sum of three components: latency, throughput, and jitter. The QoS score is computed as follows:

(7) $$QoS_{i}=\omega_{1}\times Latency+\omega_{2}\times Throughput+\omega_{3}\times Jitter$$

where $\omega_{1}$, $\omega_{2}$, and $\omega_{3}$ are the weights for latency, throughput, and jitter, respectively, representing the importance of each measure. The sum of weights $\omega_{1}$, $\omega_{2}$, and $\omega_{3}$ is always 1. The weights reflect the relative importance of each metric in ensuring reliable communication. If QoS≥0.5, the nodes are considered for further trustworthiness evaluation. The values assigned to these weights are given below.

- **$\omega_{1}=0.5$**: Given the highest weight since low latency is critical for real-time communication and network responsiveness.
- **$\omega_{2}=0.3$**: Moderately weighted because higher throughput improves performance but is not as critical as latency in determining reliability.
- **$\omega_{3}=0.2$**: Given the lowest weight since minor fluctuations in delay do not significantly impact trust unless excessive. While jitter reflects variation in packet delivery time, it is less critical in some applications of IoT. Accordingly, the weight $\omega_{3}$ assigned to jitter is the smallest value.

These values prioritize low latency for real-time responsiveness, followed by high throughput, and then minimal jitter. These weights ensure a balanced and practical trust evaluation mechanism for IoT contexts.

### 4.3. Importance of a Node

In the proposed scheme, a node’s importance within the network topology is quantified using centrality. Centrality identifies nodes that serve as key connectors or communication hubs. This plays a critical role in ensuring the efficiency, resilience, and robustness of the overall network. A node with higher centrality typically participates in more routing paths and data exchanges, which makes it both more influential and more valuable for maintaining network performance.

For a graph G=(V,E), where *V* is the set of nodes and *E* is the set of edges, the harmonic centrality of a node i∈V is defined as follows:

(8) $$Centrality_{i}=\sum_{\begin{array}{c} j\in V\\ j\neq i \end{array}}\frac{1}{d(i,j)}$$

where d(i,j) is the shortest path distance between node *i* and node *j*.

Harmonic centrality is particularly useful in our scenario because the efficiency of information dissemination and robustness of the network are important. Nodes with high harmonic centrality can quickly disseminate information to other nodes.

### 4.4. IOTA-Based Trust

To enhance the novelty of our trust evaluation framework, we introduce additional trust metrics derived from the IOTA Tangle. These metrics capture key properties of node behavior in the IOTA network, such as transaction confirmation reliability, contribution to consensus, and reattachment frequency. The following subsections define these metrics and their integration into the overall trust model.

#### 4.4.1. Transaction Confirmation Rate

Transaction Confirmation Rate (TCR) measures the proportion of a node’s transactions that are successfully confirmed in the Tangle. A higher TCR indicates a more reliable node. It is defined as follows:

(9) $$TCR=\frac{T_{c}}{T_{t}}$$

where $T_{c}$ is the number of transactions successfully confirmed, and $T_{t}$ is the total number of transactions issued by the node.

The TCR value is normalized between 0 and 1, where a value close to 1 signifies that the node’s transactions are reliably confirmed.

#### 4.4.2. Cumulative Weight Contribution

Cumulative Weight Contribution (CWC) quantifies the total weight of transactions a node has contributed to the network. This metric indicates the role of a node in securing the Tangle. It is computed as follows:

(10) $$CWC=\frac{\sum_{i=1}^{n}W_{i}}{\sum_{j=1}^{N}W_{j}}$$

where $W_{i}$ is the cumulative weight of transactions issued by the node, $W_{j}$ is the total cumulative weight of all transactions in the network, *n* is the number of transactions issued by a specific node, and *N* is the total number of transactions in the entire IOTA Tangle.

A higher CWC indicates a node’s stronger contribution to the Tangle’s consensus process.

#### 4.4.3. Reattachment Frequency

Reattachment Frequency (RF) measures how often a node reattaches its transactions due to failure in achieving confirmation. A lower RF suggests a more stable and reliable node. It is given as follows:

(11) $$RF=1-\frac{T_{r}}{T_{t}+\epsilon }$$

where $T_{r}$ is the number of reattachments attempted by the node, $T_{t}$ is the total number of transactions issued, and ϵ is a small constant to prevent division by zero. The value for ϵ in our simulations is $10^{-6}$. This value is small enough to avoid affecting results when denominators are large and large enough to prevent numerical instability when transaction counts are very low. This ensures stability while having negligible impact on the metric when $T_{t}$ is larger.

Since frequent reattachments indicate unreliable or malicious behavior, RF is designed to have higher values for more trustworthy nodes.

#### 4.4.4. Cumulative Transaction Contribution

Cumulative Transaction Contribution (CTC) represents the extent to which a node’s transactions are used in confirming other transactions. It is formulated as follows:

(12) $$CTC=\frac{\sum_{i=1}^{n}A_{i}}{\sum_{j=1}^{N}A_{j}}$$

where $A_{i}$ is the number of approvals received for the node’s transactions, and $A_{j}$ is the total number of approvals across all nodes.

Nodes with a higher CTC play a more significant role in the transaction approval process, increasing their trustworthiness.

#### 4.4.5. IOTA-Based Trust Score (ITS)

To integrate these IOTA-based metrics into a unified trust score, we define the IOTA-based Trust Score (ITS) as follows:

(13) ITS=αTCR+βCWC+γRF+δCTC

where α,β,γ,δ are weighting factors that sum to 1. These weights determine the relative importance of each metric in the trust evaluation.

We assign the following values based on the importance of each metric in trust evaluation:

- **α=0.4**: Since transaction confirmation rate is a direct indicator of a node’s reliability in handling transactions, it is given the highest weight.
- **β=0.3**: Cumulative weight contribution reflects the node’s role in transaction validation and propagation, making it the second most important factor.
- **γ=0.2**: Reattachment frequency is assigned a moderate weight to discourage excessive reattachments, which may indicate unreliability or malicious behavior.
- **δ=0.1**: Cumulative transaction contribution is given the lowest weight, as transaction volume alone does not necessarily equate to trustworthiness.

### 4.5. Trustworthiness Evaluation

The process of trustworthiness evaluation is shown in Algorithm 1. The algorithm contains functions to evaluate each metric for a node *i*. It shows the process performed by each node *j* within the communication range of a node *i* to determine local trust scores for node *i*. For *n* number of nodes within node $i^{’}$s communication range, there are *n* trust scores.

In the final phase of our trustworthiness assessment framework for IoT nodes, we employ the exponential weighting approach to calculate the overall trust score. This approach is designed to amplify the impact of each node’s performance across the metrics calculated above. This ensures that higher scores are rewarded, while lower scores are more significantly penalized. In this way, the proposed scheme provides a nuanced and sensitive analysis of each node’s overall contribution to the network’s reliability and efficiency.

The consensus algorithm is designed to operate with as few as two local trust scores, i.e., a node and one neighbor. While more scores improve robustness, the algorithm does not mandate a hard minimum beyond this. If three or more trust scores are available, the algorithm uses both the mean–median comparison to detect and smooth out outliers. If only two scores are available, the average of both is used as the agreed trust score. If a node has no neighbors in a temporarily disconnected scenario, the trust score is marked as undefined and is excluded from trust-based operations until more data are available.
**Algorithm 1:** Optimized Trustworthiness Evaluation**Input:** $Message_{i}$, IOTA_transactions**Output:** Node trust value  1:**for** Each node *i* **do**  2:    $Reliability_{i}\leftarrow computeReliability\left(T_{i},bit\;errors,bits\;transferred\right)$  3:    $QoS_{i}\leftarrow computeQoS\left(T_{i},T_{i,j},data\;transmitted\right)$  4:    $Centrality_{i}\leftarrow computeCentrality\left(i,Graph\right)$  5:    $IOTA\_trust_{i}\leftarrow computeIOTATrust\left(i,IOTA\_transactions\right)$  6:    **if** $Reliability_{i}\geq 0.5$ AND $QoS_{i}\geq 0.5$ **then**  7:        **for** Each node *j* within range **do**  8:           Send $Message_{i}$ to *j*  9:           $trust_{i}\leftarrow computeTrust\left(Reliability_{i},QoS_{i},Centrality_{i},IOTA\_trust_{i}\right)$10:        **end for**11:    **else**12:        $trust_{i}\leftarrow 0$                                                                   ▹ Untrusted node13:    **end if**14:**end for**

The local trust score for each node is computed using an exponential function applied to the key metrics: reliability, QoS, and harmonic centrality. The formula is as follows:

(14) $$\mathrm{local}\_\mathrm{trust}=\sum_{i=1}^{n}w_{i}\times e^{\left(s_{i}\right)}$$

where:

- $s_{i}$ represents the normalized score of each metric.
- $w_{i}$ denotes the weight assigned to each metric, reflecting its importance in the overall assessment of trustworthiness.
- The exponential function $e^{\left(s_{i}\right)}$ is applied to each score, enhancing the distinction between varying performance levels.

The ITS score is then combined with the trust evaluation model to compute the Final Trust Score (FTS):

(15) FTS=(λ)ITS+(1−λ)local_trust

where λ controls the contribution of IOTA-based metrics versus traditional trust factors such as reliability, QoS, and centrality.

To compute node $i^{\prime }$s trust score, a consensus mechanism is given in Algorithm 2. In the algorithm, the trust score computed by node *j* for node *i* is denoted as $local\_trust_{j}$. Each node *i* initializes an empty list to store trust scores from all nodes *j* within its communication range. Each node *j* computes its local trust value for node *i* using the computeTrustValue function and appends it to the list of trust scores for node *i*. The consensusAlgorithm function sorts the collected trust scores. It then calculates the median and mean of these scores. If the difference between the mean and median is below the consensus threshold, the mean is considered as the agreed trust score. Otherwise, the median is used. The agreed trust score for node *i* is broadcasted to all nodes within its communication range.
**Algorithm 2:** Consensus Mechanism for Trust Scores**Input:** Trust scores from nodes *j* for node *i***Output:** Agreed trust score for node *i*  1:**for** Each node *i* **do**  2:    Initialize trust_scores[i]←[]  3:    **for** Each node *j* within communication range of *i* **do**  4:        $trust_{j}$←computeTrustValue($Rel_{j}$, $QoS_{j}$, $Centrality_{j}$, $IOTA\_trust_{j}$)  5:        Append $local\_trust_{j}$ to trust_scores[i]  6:    **end for**  7:    $agreed\_trust_{i}$←consensusAlgorithm (trust_scores[i])  8:    Broadcast $agreed\_trust_{i}$ to all nodes *j* within communication range  9:**end for**10:**function**consensusAlgorithm(trust_scores)11:    sorted_scores← sort(trust_scores)12:    median← median(sorted_scores)13:    mean← mean(sorted_scores)14:    **if** mean−median<threshold **then**15:          agreed_trust←mean16:    **else**17:          agreed_trust←median18:    **end if**19:    **return** agreed_trust20:**end function**

### 4.6. Distributed Storage of Trust Values

In this phase, we utilize the IOTA Tangle to securely store the agreed trust scores of all nodes in a distributed and immutable manner. This ensures that no node in the network presents a fake trust value for malicious intention. It also stores the values in a distributed fashion, so that any other node in the network can access and verify the trust scores of another node conveniently. We configure the IOTA SDK (https://github.com/iotaledger/iota-sdk (accessed on 25 May 2025)) for the Shimmer network by setting up the client libraries and establishing a connection to the Shimmer testnet. The integration of the IOTA ledger involves the following steps.

1. Transaction Creation:
  - Encoding the agreed trust score data into a suitable format for IOTA transactions. This ensures that the data payload adheres to the protocol’s requirements.
  - Creating a transaction object in the IOTA SDK to embed the encoded trust score data into the transaction message.
  - Attaching the metadata about the respective node to the transaction to facilitate easy retrieval and identification.
2. Transaction Submission:
  - Submitting the transaction to the IOTA network via the IOTA SDK client.
  - Monitoring the network response to ensure that the transaction is successfully broadcasted and confirmed by the network.
3. Verification and Confirmation:
  - Verifying the transaction status using the transaction ID provided by the IOTA network.
  - Confirming the immutability of the trust score data by ensuring it is included in a milestone, making it a permanent part of the IOTA ledger.
4. Broadcasting and Retrieval:
  - Broadcasting the transaction ID to the stored trust score to all nodes within the network.
  - Nodes can retrieve and verify the trust score directly from the IOTA ledger using the provided transaction ID, ensuring transparency and trust in the stored trust data.

In the proposed framework, each node’s trust update is packaged as an individual transaction, signed, and submitted directly to the Tangle. There is no logical or temporal dependency on block creation. Finality is achieved once the transaction is referenced by a milestone. This ensures that the transaction becomes an immutable part of the ledger. This lightweight, transaction-first model aligns with the low-latency and high-throughput requirements of IoT trust management. Moreover, the integration of the IOTA Tangle ensures that trust scores are securely stored in an immutable, decentralized manner. This enhances the reliability, security, and transparency of the trust evaluation framework, providing a robust solution for managing trust in IoT environments.

## 5. Results and Discussion

In this section, we evaluate the proposed scheme within IoT networks.

### 5.1. Simulation Setup

The simulations have been performed using an Ubuntu 22.04 operating system on NS3 (https://www.nsnam.org (accessed on 25 May 2025)) (version 3.36.1) as the network simulator. To evaluate the proposed scheme, a comprehensive simulation has been performed. The simulation environment has been configured to add nodes equipped with IoT sensors. Table 2 provides a summary of the simulation parameters.

Nodes collect QoS metrics and reliability data over sliding observation windows of 10 s, which are repeated at intervals of 60 s. The 10 s window is sufficient to capture recent performance trends without causing excessive computation or communication overhead. Nodes perform trust computations only at each interval of 60 s, which reduces the frequency of evaluations while maintaining responsiveness to behavioral changes. To avoid reliance on outdated data, old observations are discarded after each trust computation cycle. This ensures that trust scores reflect the most recent behavior without cumulative bias.

### 5.2. λ Sensitivity Analysis

**Figure 2:**
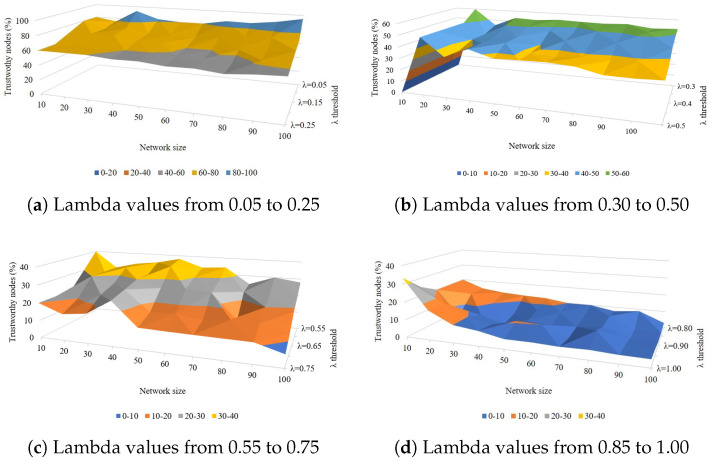
Effect of different lambda threshold values on node trustworthiness.

The proposed scheme uses a trust threshold called λ to determine the trustworthiness of a node. This threshold provides a trade-off between number of trustworthy nodes and privacy. Figure 2 shows that there is an inverse relationship between λ values and the number of trustworthy nodes. Therefore, when the information is more sensitive, a higher threshold value is selected in order to only select the nodes with highest final trust score. However, when the information is not very sensitive, lower values of λ return many trustworthy nodes, contributing to network efficiency.

It has been noted that λ values between 0.25 and 0.50 offer an optimal number of trustworthy nodes for a network of 10–100 nodes. The wide range of λ values (0.05 to 1.00) provides flexibility in adjusting the strictness of trust evaluation. This allows for fine-tuning based on specific network requirements or security needs.

Our trust evaluation method demonstrates a nuanced approach to assessing node trustworthiness. By adjusting the λ parameter, we can control the balance between network security and inclusivity. The method appears to be adaptable to different network sizes, which is crucial for real-world applications where network scale can vary significantly. These conclusions highlight the flexibility and effectiveness of our trust evaluation method in various scenarios.

### 5.3. IOTA Block Generation and Retrieval Costs

**Figure 3:**
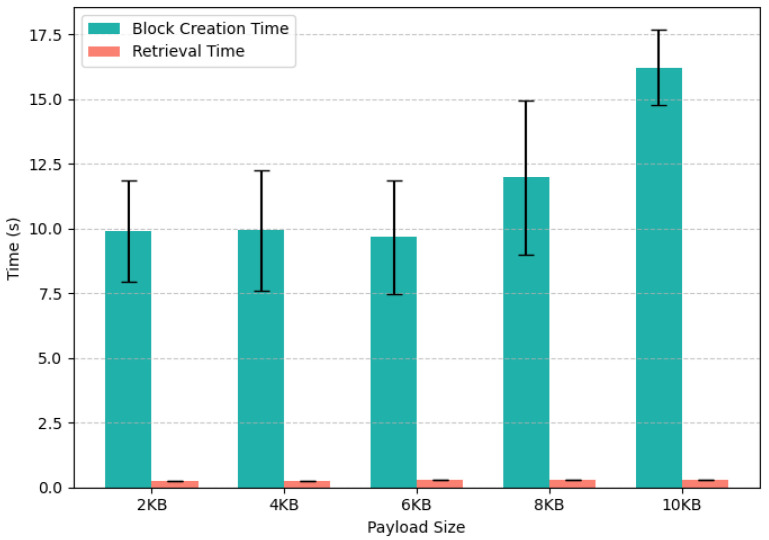
The cost of generating and reading blocks on IOTA for different payload sizes.

For this experiment, 1000 iterations have been recorded. Figure 3 shows the results of the experiment. The block generation cost does not vary significantly for payload sizes from 2 Kb to 8 Kb, staying between 11.67 s and 13.97 s. However, when the payload size increases to 10 Kb, the block generation cost slightly increases from 13.97 s to 18.83 s. IOTA is utilized in the proposed scheme to securely store trust values. The trust values are saved in JSON format with three entries: node ID $ID_{node}$, previous average trust score $trust_{t-1}$, and current trust score $trust_{t}$. These data result in a small payload size. However, we experiment for up to 10 Kbs of payload size. It is noted that the block generation cost stays within reasonable bounds.

Similarly, the cost of block reading stays consistent across all payload sizes, as shown in Figure 3. The block retrieval time for a 2 Kb payload size is 1.52 s, and that of 10 Kb stays at 1.87 s. This shows that the nodes retrieving trust values from the IOTA Tangle do not require extensive computing resources. This makes the proposed scheme cost-efficient in terms of resource consumption for different operations.

The error bars in Figure 3 represent the variability in the measured times across 1000 iterations, quantified here as the standard deviation. For block retrieval times, which range from approximately 0.25 s to 0.29 s depending on the payload size, the corresponding error rates are extremely low, between 0.00044 s and 0.00068 s. This demonstrates highly consistent retrieval performance. Similarly, the block creation times vary from roughly 9.67 s to 11.97 s, with associated errors between 11.84 s and 14.93 s. Although these error values for block creation appear large relative to the averages, they reflect occasional spikes in processing time due to network latency and computational overhead during block formation. Together, these error bars provide a clear depiction of the stability and reliability of both block creation and retrieval operations under varying payload sizes. This confirms that the proposed trust evaluation scheme maintains predictable performance despite natural system fluctuations.

#### FTS Computation Cost

**Figure 4:**
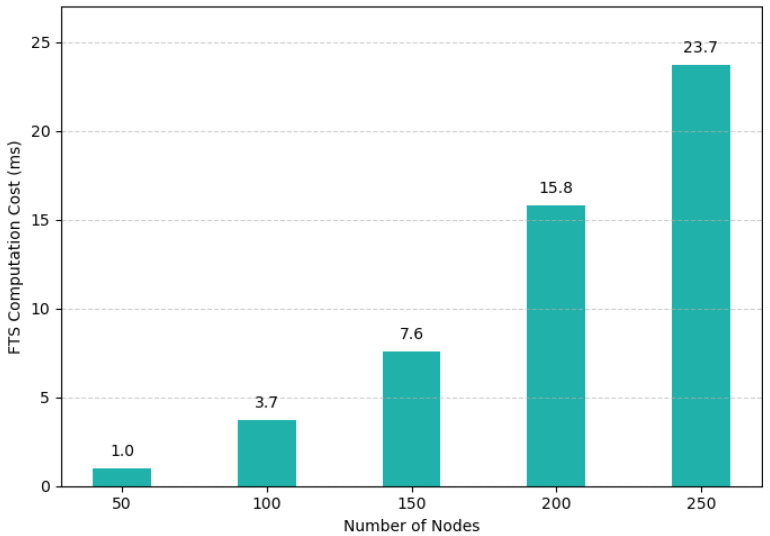
The cost to compute FTS when moving from a small network (50 nodes) to a large network (250 nodes), reported in ms.

To evaluate the scalability of the proposed trust framework, we conducted experiments measuring the computational cost of calculating the FTS across increasing network sizes. Figure 4 presents the average time taken to compute the FTS per node as the number of nodes increases from 50 to 250. This trend indicates that while computation time increases as more nodes contribute to trust aggregation, the cost remains well below 25 ms, even for 250 nodes.

The increase in cost is attributed primarily to the higher number of neighbor interactions and trust score inputs and additional local consensus operations required to ensure robustness in larger peer sets. Despite this growth, FTS computation remains lightweight in the context of IoT operations. Since trust is evaluated at intervals (every 60 s), even the highest recorded cost (23.7 ms) represents less than 0.04% of a trust cycle.

These results affirm that the proposed framework offers excellent scalability in terms of computational overhead, even as the network scales toward hundreds of devices.

### 5.4. Performance Comparison

We compare the performance of the proposed scheme with the scheme proposed by Wang et al. [33], Chen et al. [39], and Mo et al. [40]. Apart from evaluating the malicious node detection rate, we compare the proposed scheme based on throughput, energy consumption, and end-to-end delay. Throughput reflects data integrity and routing success under trust-guided paths. Energy consumption is critical for evaluating resource efficiency, especially in battery-operated sensor nodes. End-to-end delay captures the responsiveness of the trust mechanism in facilitating timely communication. Together, these metrics provide a comprehensive assessment of both the trust system’s security and its operational impact.

#### 5.4.1. Malicious Node Detection Accuracy

**Figure 5:**
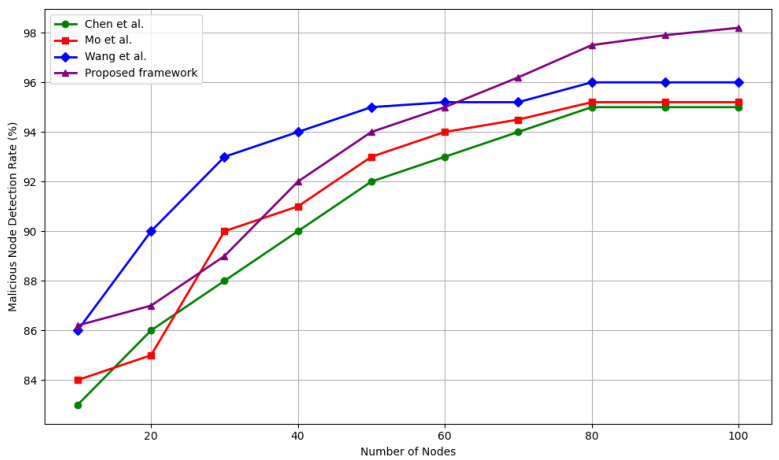
The detection rate of malicious nodes against different network sizes as compared to Wang et al. [33], Chen et al. [39], and Mo et al. [40].

Detection accuracy is defined as the percentage of adversarial nodes correctly identified and isolated by the system. As illustrated in Figure 5, the proposed framework consistently outperforms existing approaches across all evaluated scenarios. In low adversary densities, i.e., 5–15 malicious nodes, all methods show comparable accuracy, with our framework achieving up to 89%, which is within 3% of the best-performing baseline. However, as the proportion of malicious nodes increases, the advantage of our method becomes more prominent.

In medium-scale attack scenarios, i.e., 20–30 malicious nodes, the proposed method achieves up to 95% detection accuracy, marking a +2.1% gain over Wang et al. [33], +1.0% over Mo et al. [40], and +2% over Chen et al [39]. This improvement is due to the integration of ledger-anchored behavior metrics (transaction confirmation rate and reattachment frequency), which enhance the distinction between honest and dishonest activity beyond traditional network-level observations.

Under large-scale attacks, i.e., 35–50 malicious nodes, our framework achieves detection rates of up to 98.2%, compared to 96.0% for Wang et al. [33], and 95.2% for both Mo et al. [40] and Chen et al. [39]. This translates to a +2.2% absolute improvement over the best baseline (Wang et al.) [33] and a +3.0–3.2% gain over the others. These gains highlight the robust scalability of our trust mechanism under increasing adversarial influence.

#### 5.4.2. Malicious Node Detection vs. Adversarial Density

**Figure 6:**
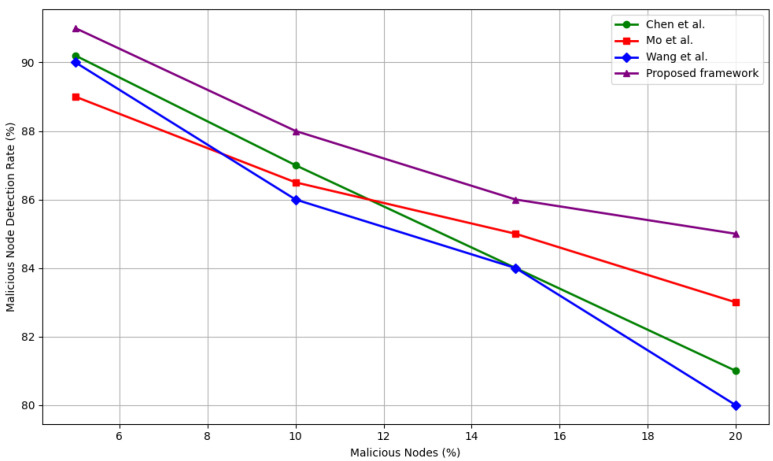
Detection rate of malicious nodes against adversarial density as compared to Wang et al. [33], Chen et al. [39], and Mo et al. [40].

To evaluate robustness under varying levels of adversarial infiltration, we compare the detection accuracy of our framework with Chen et al. [39], Mo et al. [40], and Wang et al. [33] under increasing percentages of malicious nodes (5%, 10%, 15%, and 20%). Figure 6 presents the comparative results.

In low adversary settings (5%), all models perform competitively, with detection rates ranging between 88 and 90%. Our framework achieves 90%, matching or exceeding all baselines. As adversary density increases, the gap between traditional models and the proposed approach becomes more evident.

At 10–15% adversarial presence, while Chen et al. [39] and Wang et al. [33] experience a 3–6% decline in detection accuracy, our framework maintains a relatively stable performance at 87–85%, indicating superior resistance to moderate-scale trust disruption. At the 20% mark, our method outperforms all baselines, achieving 85% detection—4% higher than Wang et al. [33], 4% higher than Chen et al. [39], and 2% higher than Mo et al. [40].

This stability under pressure is attributed to our model’s use of cross-layer trust signals, including both communication-based metrics and immutable behavioral logs recorded on the IOTA Tangle. Malicious nodes struggle to generate legitimate Tangle contributions. This leads to rapid and consistent detection, even when attackers outnumber defenders.

#### 5.4.3. Throughput Scalability with Network Size

**Figure 7:**
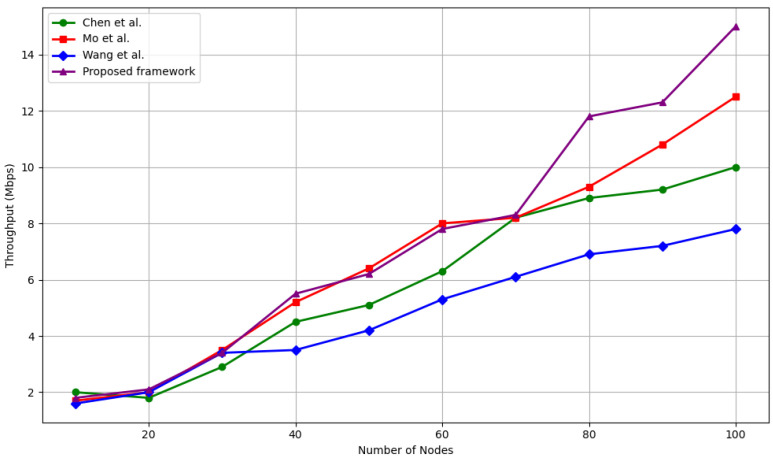
Throughput comparison among Wang et al. [33], Chen et al. [39], and Mo et al. [40] (Mbps).

Throughput performance has been assessed as the number of nodes scaled from 10 to 100, with comparisons against Wang et al. [33], Chen et al. [39], and Mo et al. [40]. The results are shown in Figure 7.

At small scales, i.e., 10–30 nodes, all models perform similarly, with throughput ranging from 1.6 to 3.5 Mbps. The proposed framework maintains parity here, achieving 3.4 Mbps at 30 nodes. However, as the network grows beyond 40 nodes, our framework demonstrates a clear throughput advantage, highlighting its scalability.

At 80 nodes, our system achieves 11.8 Mbps, which is:

- +2.5 Mbps (26.6%) higher than Mo et al. [40]
- +4.9 Mbps (71%) higher than Wang et al. [33]
- +2.9 Mbps (32.5%) higher than Chen et al. [39]

At full scale, i.e., 100 nodes, the proposed method reaches 15 Mbps, outperforming Mo et al. [40] by +2.5 Mbps, and exceeding Wang et al. [33] and Chen et al. [39] by nearly +7 Mbps, representing a 93% and 50% improvement, respectively.

This throughput gain is a direct result of the trust-guided routing and ledger-verifiable trust validation, which reduces misrouting and packet retransmission due to malicious nodes. The absence of centralized decision-making also allows the network to avoid bottlenecks and maintain high data flow as it scales.

#### 5.4.4. Energy Consumption Analysis

**Figure 8:**
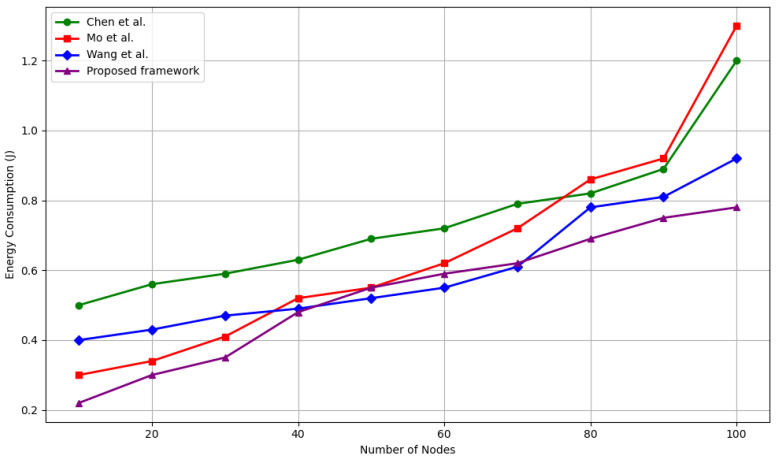
Energy consumed by Wang et al. [33], Chen et al. [39], and Mo et al. [40] (J).

Energy efficiency is critical in IoT networks, where many nodes are battery-powered and operate in constrained environments. Figure 8 compares the average energy consumption per node of the proposed framework against Chen et al. [39], Mo et al. [40], and Wang et al [33]. as the network scales from 10 to 100 nodes.

Our framework consistently consumes less energy than all baselines across the entire range. At 10 nodes, it achieves an average consumption of 0.22 J, representing a 26.7% reduction compared to Wang et al. [33] (0.3 J), 26.7% compared to Mo et al. [40] (0.3 J), and 56% compared to Chen et al. [39] (0.5 J). This energy advantage persists and grows with network size.

At 100 nodes, our framework uses 0.78 J per node, which is:

- 40% less than Wang et al. [33] (1.3 J)
- 40% less than Mo et al. [40] (1.3 J)
- 35% less than Chen et al. [39] (1.2 J)

This reduction is primarily due to our lightweight trust evaluation computations and avoidance of cluster-based overheads inherent in some baselines. Additionally, our use of local, asynchronous consensus combined with efficient IOTA ledger operations minimizes redundant transmissions and retransmissions, which conserves battery life.

#### 5.4.5. End-to-End Delay Analysis

**Figure 9:**
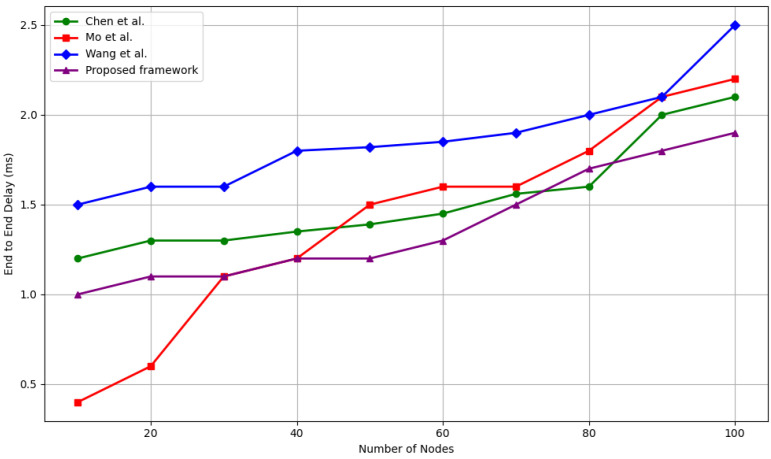
End-to-end delay comparison among Wang et al. [33], Chen et al. [39], and Mo et al. [40] (ms).

End-to-end delay directly impacts the responsiveness and quality of service in IoT applications. Figure 9 shows the delay measurements for the proposed framework and compared methods over varying network sizes.

At smaller network sizes, i.e., 10–30 nodes, our framework achieves delays of between 1.0 ms and 1.1 ms, which is comparable to or better than all baselines. Notably, at 10 nodes, it is 20% faster than Chen et al. [39] (1.2 ms) and 60% faster than Wang et al. [33] (1.5 ms).

As network size grows to 100 nodes, our framework maintains an average delay of 1.9 ms, which is:

- 13% lower than Chen et al. [39] (2.1 ms)
- 14% lower than Mo et al. [40] (2.2 ms)
- 24% lower than Wang et al. [33] (2.5 ms)

The relatively low delay is attributed to our distributed trust evaluation approach, which avoids delays caused by hierarchical or cluster-based leader election protocols. Additionally, IOTA’s asynchronous transaction validation allows for trust updates without blocking network traffic.

### 5.5. Informal Security Analysis

The proposed trust evaluation framework resists against the attacks listed under the attacker model.

#### 5.5.1. Sybil Attack

In a Sybil attack, an adversary spawns multiple identities to flood the trust system. The purpose is to artificially inflate its own trust score. IOTA-based trust metrics prevent the nodes from gaining high trust maliciously. Each node must issue verifiable transactions with confirmation. Repeated transaction failures and reattachment attempts by fake identities lower RF scores.

#### 5.5.2. Self-Promotion Attack

In this type of attack, a malicious node attempts to inflate its own trust score through fake or repeated transactions. This is mitigated by using the metrics TCR and RF. TCR exposes nodes issuing unverifiable or reattached transactions and RF penalizes nodes that constantly reissue transactions for visibility.

#### 5.5.3. Bad-Mouthing Attack

While calculating indirect trust scores, malicious nodes give deliberately low ratings to honest peers to reduce their trustworthiness in a bad-mouthing attacks. This is prevented through median-based consensus, as given in Algorithm 2. Median-based consensus smooths out extreme values. Because of the diverse feedback, honest nodes provide positive scores. This dilutes bad-mouthing attempts. Nodes with high reliability/QoS/ITS maintain higher influence in consensus weighting.

#### 5.5.4. Collusion Attack

In a collusion attack, the attacker isolates a victim node by controlling all its neighboring connections and feeding it false trust scores. This attack is prevented in this study because the proposed framework does not rely on one peer. Trust is computed locally by every node within the range. Even if one node is isolated, its trust score must be accepted by others. Moreover, final trust scores are publicly verifiable on the Tangle.

#### 5.5.5. Resource Depletion Attack

In this attack, the attackers flood the network with trust-related traffic to drain resources. This is prevented by ensuring lightweight scoring. Local trust evaluations use simple equations, which reduce CPU strain. Additionally, there is no centralized trust node, which ensures there is no bottleneck and single point of overload. Nodes evaluate and store trust independently, avoiding synchronized peaks in computation.

## 6. Conclusions

We propose a decentralized trust evaluation framework for IoT networks using IOTA ledger technology. Our approach addresses the critical vulnerabilities of centralized trust models, such as single points of failure and privacy breaches. By decentralizing the trust assessment process and integrating both direct and indirect trust metrics, we ensure comprehensive and reliable evaluation of trustworthiness. The use of IOTA for storing trust values guarantees the immutability and security of trust data, preventing any node from transmitting fake values. Our experimental results demonstrate significant improvements in energy efficiency, scalability, and end-to-end delay compared to traditional centralized models. The decentralized nature of our framework enhances the overall security posture of IoT environments, making them resilient to various cyber-attacks. Additionally, the secure and immutable storage of trust scores ensures data integrity and confidentiality, which are paramount in maintaining the reliability of IoT systems.

In future, this framework can be further strengthened through integration with emerging AI technologies, particularly large language models (LLMs) and edge intelligence. AI agents can dynamically adjust trust weights ($\lambda ,\rho ,\omega_{1}-\omega_{3}$) in real time based on contextual factors such as environmental conditions, device behavior, and threat detection. Additionally, LLMs could be leveraged for intelligent anomaly detection, trust decision explanations, and federated trust summarization across large IoT ecosystems. This synergy could pave the way for adaptive, explainable, and self-healing trust frameworks in future smart cities, autonomous systems, and decentralized AI infrastructures. 

## Figures and Tables

**Table 1:** Summary of the recent literature.

Trust Model	Storage	Ledger/Platform	Metrics Used	Consensus/Update Mechanism	Attack Resilience
[21]	Local	None	User satisfaction, node recommendations	Weighted sum with context adjustment	Low (context spoofing possible)
[22]	Local	None	Cross-validation between UAV and vehicle data	Distributed reputation propagation	Medium (dependent on honest majority)
[23]	Local	None	Honesty, energy level, recommendations	Continuous updates with neighbor reports	Low (lacks attack modeling)
[24]	Local	None	Data quality, security, referral trust	Sliding time window-based updates	Medium (filtering included)
[25]	Blockchain-based	Ethereum	Service quality, history of data interactions	PoW-based consensus (Ethereum)	Medium (secure but resource-intensive)
[26]	Local	None	Community leader ratings, node reputation	Centralized cluster aggregation	Medium (leader compromise risk)
[27]	Blockchain	Hyperledger	Behavioral history, trust contracts	Smart contracts (PoA)	High (ledger-backed trust)
[28]	Blockchain	Custom DLT	Routing behavior, transport delay, consistency	Decentralized updates + DLT logs	Medium–high (immutable audit trail)
[29]	Verifiable logs	Blockchain	Historical baseline deviation	Cryptographic + statistical verification	High (tamper-evident logs)
Proposed scheme	Fully decentralized	IOTA Tangle	Reliability, QoS, centrality, Tangle-based trust	Local mean–median consensus	High (Sybil, collusion, eclipse resilience)

**Table 2:** Simulation parameters.

Modules	Parameters	Values
Operating system	Ubuntu	22.04.1
		NS3 (ns-3.36.1)
Network topology	Node placement	Random disc position (radius = 300 m)
	Traffic pattern	Constant Bit Rate (CBR)
	Communication range	250 m
	Number of nodes	50-250
	Mobility model	ConstantPositionMobilityModel
Node mobility	Base station mobility	Constant position
	Node mobility	Constant position
	Position allocation	Random disc position allocator
Propagation	Packet size	1000 bytes
	Constant rate	100 Kbps
	Physical layer modulation and coding scheme	DsssRate11Mbps
	Data rate	5 Mbps
	Propagation loss model	Friis propagation loss model
Wi-Fi configuration	Wi-Fi standard	802.11b
	Transmit power start	20.0 dBm
	Transmit power end	20.0 d
	Receiver gain	30 dB

## Data Availability

Data is contained within the article. The NS-3 simulation code used to generate the results is available from the authors upon request.
